# Whole genome of *Lasiodiplodia theobromae*, *Lasiodiplodia euphorbiaceicola,* and *Lasiodiplodia brasiliensis* isolates associated with Mango Dieback and stem-end rot in Côte d’Ivoire

**DOI:** 10.1128/mra.01500-25

**Published:** 2026-07-24

**Authors:** Yéfoungnigui S. Yeo, Alexis Dereeper, Daouda Kone, Diana Fernandez

**Affiliations:** 1 Wascal/African Center of Excellence in Climate Change, Biodiversity and Sustainable Agriculture, University Félix Houphouët-Boigny, Abidjan, Côte d’Ivoire; 2 UMR PHIM (Plant Health Institute), University of Montpellier, IRD, CIRAD, INRAE, Institute Agro 27037, Montpellier, France; University of California Riverside, Riverside, California, USA

**Keywords:** *Lasiodiplodia* spp., *Mangifera indica*, mango dieback, mango stem-end rot, genomic resources, phylogenetic analysis

## Abstract

Mango productivity is threatened by dieback and fruit rot from *Lasiodiplodia* spp. (*Botryosphaeriaceae* fungal family). We report high-quality genomes of *Lasiodiplodia theobromae*, *Lasiodiplodia euphorbiaceicola*, and *Lasiodiplodia brasiliensis* from Côte d’Ivoire, enhancing genomic resources for comparative and pathogenicity studies of these fungal plant pathogens.

## ANNOUNCEMENT

Mango (*Mangifera indica* L.) productivity is severely constrained by Mango Dieback (MD), a vascular disease characterized by chlorosis, dieback of twigs and branches, gommosis, and tree mortality (1, 2). In addition, mangoes may suffer post-harvest stem-end rot (SER) marked by necrotic lesions at the fruit stem end, leading to over 20% of fruit losses (3, 4). Members of the *Botryosphaeriaceae* fungal family, particularly *Lasiodiplodia* spp., have been consistently responsible for MD and SER outbreaks in mango production (4, 5). Whole-genome sequences were produced for *Lasiodiplodia theobromae* strains isolated from grapevine (6), cacao (7), citrus (8), and peach (9), for *Lasiodiplodia citricola* from Populus (10), and for *Lasiodiplodia iranensis* from peanut (11). However, no genome is yet published for *Lasiodiplodia* spp. isolates from mango MD or SER.

In this study, we sequenced and assembled the genomes of three *Lasiodiplodia* spp. isolates 01FSyA8 (9°31′16.40″N, 7°36′15.93″W), 30BrSyA5 (9°32′45″N, 5°25′55″W), and LaRA1 (9°22′08″N, 5°42′59″W), collected from symptomatic mango trees in Côte d’Ivoire in 2020 as described in reference 12. Genomic DNA was extracted from mycelia of 5-day-old fungal cultures grown on potato dextrose agar as described in a previous study (13). Prior to genome sequencing, species-level identification of the isolates was conducted using the internal transcribed spacer (ITS) and translation elongation factor *1-alpha* (*tef1-α*) markers. BLASTn analyses revealed 100% sequence identity with *L. theobromae* (01FSyA8), *Lasiodiplodia euphorbiaceicola* (30BrSyA5), and *Lasiodiplodia brasiliensis* (LaRA1), respectively. The ITS and *tef1-α* sequences were deposited in the NCBI GenBank under the following accession numbers: OR264287 (ITS) and OR271546 (*tef1-α*) for 01FSyA8; OR264288 (ITS) and OR271548 (*tef1-α*) for 30BrSyA5; and OR264286 (ITS) and OR271540 (*tef1-α*) for LaRA1.

Sequencing libraries were prepared via the Illumina TruSeq Nano DNA Kit and sequenced on the NovaSeq 6000 platform using 150 bp paired-end reads with 50× coverage. Raw reads were quality-filtered with *fastp* (14), and genome sizes were estimated by 15-mer analysis. Assembly was performed using Assembly Abyss V2.2.1 (15), with subsequent gap closure via TGS-GapCloser v1.12 (16). Contigs shorter than 500 bp were excluded.

**Fig 1:**
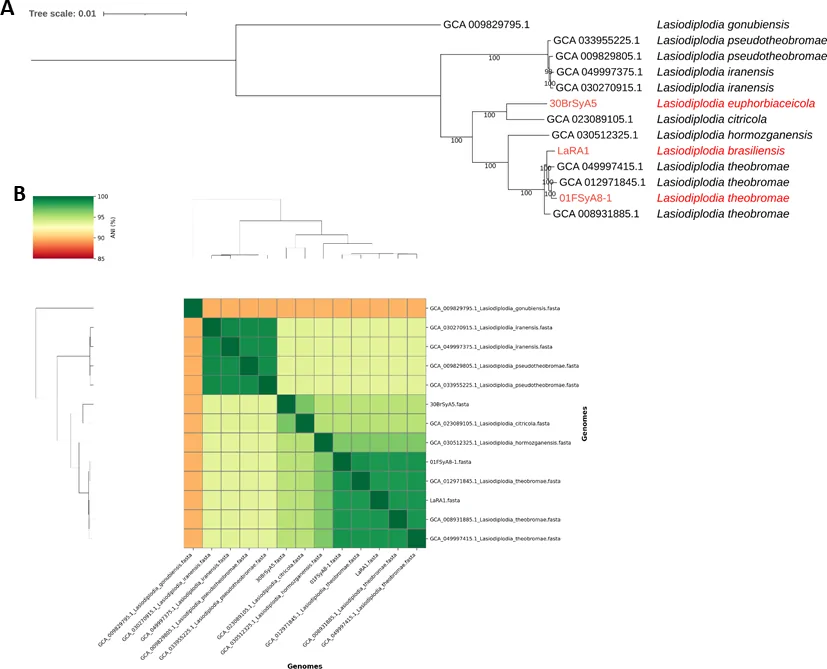
Phylogenetic and nucleotide similarity analysis of three newly sequenced genomes within the genus *Lasiodiplodia*. (**A**) SNP-based phylogenetic relationships among publicly available genomes of the genus *Lasiodiplodia* and three newly sequenced genomes. A core genome SNP alignment was generated using Parsnp (18), with *L. theobromae* GCA_012971845.1 as the reference. Maximum-likelihood phylogenetic inference was performed using RAxML (19) under the GTRCATX model. Branch support values correspond to 100 bootstrap replicates. The tree was edited with iTOL 7.3 (21). Newly sequenced genomes are highlighted in red. (**B**) ANI analysis was performed using fastANI software (20) between publicly available genomes of the genus *Lasiodiplodia* and three newly sequenced genomes.

Resulting assemblies comprised between 109 and 176 scaffolds per isolate, with N50 values ranging from 1,150,946 bp (30BrSyA5) to 1,682,829 bp (LaRA1) and a GC content of 54.31%–54.81% (Table 1). Assembly completeness, assessed using BUSCO v5.3.2 (17) against fungi_odb10lineages (758 genes), exceeded 99% for all genomes. Phylogenetic relationships were assessed among publicly available genomes of the genus *Lasiodiplodia* and the three newly sequenced genomes. A core genome SNP alignment was generated using Parsnp (18), with *L. theobromae* GCA_012971845.1 as the reference. Maximum-likelihood phylogenetic inference was performed using RAxML (19) under the GTRCATX model. An average nucleotide identity (ANI) analysis was also performed using fastANI software (20). These indicated that the 01FSyA8 (*L. theobromae*) genome closely grouped with *L. theobromae* genomes in the same clade with LaRA1 (*L. brasiliensis*) (Fig. 1). In contrast, the 30BrSyA5 (*L. euphorbiaceicola*) genome grouped in another clade, close to *L. citricola*. These genome drafts of *L. theobromae*, *L. euphorbiaceicola*, and *L. brasiliensis* enrich the genomic resources for *Lasiodiplodia* spp. and provide a valuable foundation for future comparative genomics, pathogenicity studies, and fungal disease management strategies in mango.

**TABLE 1:** Genome statistics of three *Lasiodiplodia* species isolated from mango in Côte d’Ivoire

Species names	Isolate	Reads number	Coverage depth (×)	Assembly				% of complete	BUSCO^*a*^ analysis			
				Total length of sequence (Mb)	Contigs ≥ 500 bp	N50 (Mb)	GC content (%)		% of complete and single copy	% of complete and duplicated	% of fragmented	% of missing
*L. theobromae*	01FSyA8	15,158,241	50	43.59	116	1.67	54.81	99.2	98.5	0.7	0.5	0.3
*L. euphorbiaceicola*	30BrSyA5	13,326,857	40	43.7	176	1.15	54.31	99.3	98.8	0.5	0.5	0.2
*L. brasiliensis*	LaRA1	15,158,241	42	43.41	109	1.68	54.64	99.2	98.7	0.5	0.5	0.3

^a^ BUSCO here is based on ”fungi_odb10” set of 758 common fungal genes.

## Data Availability

This Whole-Genome project has been deposited in the European Nucleotide Archive (ENA) under the study: PRJEB101385 with accession numbers SAMEA120420990 (01FSyA8-1), SAMEA120420991 (30BrSyA5), and SAMEA120420992 (LaRA1). Assembly names are GCA_977013645.2 (01FSyA8-1), GCA_977013655.2 (30BrSyA5), and GCA_977013665.2 (LaRA1). Data needed to reconstruct the tree are archived at https://doi.org/10.5281/zenodo.18878086.
